# Knowledge, Attitude and Practices (KAP) Survey on Water, Sanitation and Hygiene in Selected Schools in Vhembe District, Limpopo, South Africa

**DOI:** 10.3390/ijerph10062282

**Published:** 2013-06-04

**Authors:** Jerry E. Sibiya, Jabulani Ray Gumbo

**Affiliations:** Department of Hydrology and Water Resources, University of Venda, Thohoyandou 0950, South Africa; E-Mail: sibiya.jerry@yahoo.com

**Keywords:** handwashing, water and sanitation, personal hygiene, water supply, waterborne diseases

## Abstract

This study assessed the knowledge, attitude and practices (KAP) of learners on issues related to water, sanitation and hygiene in selected schools in Vhembe District, South Africa. The methodology relied on a questionnaire, an inspection of sanitary facilities and discussion with the school authorities. The data was analyzed using the Statistical Package for Social Science. The study revealed that the level of knowledge about waterborne diseases was relatively high (76.7 ± 1.75%), but knowledge on transmission routes was inadequate. The majority of the respondents had no knowledge when it comes to water-based diseases and their prevention (78.4 ± 1.71%).The attitude and practice on hygiene was also found to be high (91.40 ± 1.16%). Some schools from the urban area had proper handwashing facilities, but there was no soap available. The borehole water quality for rural schools appeared clear, but the microbial quality was unknown. The water supply and sanitation facilities were inadequate in rural schools, with no handwashing areas and no sanitary bins for girls. Some schools had toilets with broken doors which did not offer privacy. The only water tap, located at the centre of the school premises, was not enough for the whole school community.

## 1. Introduction

During the last decade, South Africa has achieved mixed success on the provision of safe water supply and sanitation to rural communities [1]. At the household and school level there are concerns about the quality and use of these water and sanitation facilities. A study conducted by the Water Research Commission in 2002 indicated that rural areas suffered massive backlogs regarding the provision of adequate sanitation [2]. It also indicated that access to adequate sanitation reduced the incidence of disease and brings relative comfort and ease to the daily routine of toilet use, thereby enhancing the quality of life.

Improving the access to safe drinking water and adequate sanitation, as well as promoting good hygiene, are key components in the prevention of diarrhoea. A recent report by the World Health Organization in collaboration with UNICEF indicated that in 2006 (the latest year for which data is available), an estimated 2.5 billion people were lacking improved sanitation facilities. Moreover, nearly 1 in 4 people in developing countries were practicing open defecation [3].

Even though sanitation delivery in South Africa has increased sharply since the early 2000, with about 17,000 sanitation units being delivered a year (excluding urban sanitation provided under the national housing programme) [4]. Nevertheless, the knowledge, attitude and practices still remain as major challenges facing our communities at large. This is caused by the fact that, even if the infrastructure is there, there is no guarantee that people will use it accordingly all the times.

In addition to the provision of safe community water supply and sanitation services, there is a need for education on hygiene [5,6]. This is important as it will ensure the correct and proper use of the services long after the technical consultants have left. That is where behavior and attitude become important in the subject of water supply and sanitation. The Millennium Development Goal (MDG) number 7, for the year 2015, is aimed at reducing the proportion of people without sustainable access to safe drinking water and basic sanitation facilities by half, focusing mostly on the provision of infrastructure to meet the demands of communities in developing countries [3]. This focus has resulted in an evaluation of existing methods to identify those suitable for these activities. However, this shift in focus failed to address the manner in which people’s knowledge, attitudes and practices may contribute towards the sustainability of water supply and sanitation facilities.

UNICEF has published extensive material on school sanitation and hygiene intended at facilitating that learners be agents for change as they live within the community [7]. This is achieved by evaluation of the hardware aspects, such as the physical infrastructure, sanitation facilities at schools and the availability of safe water. The softer side includes the provision of knowledge on hygienic methods followed by their continued use (practices) at the schools. The ultimate goal is the reduction in water and sanitation related diseases otherwise if the facilities are rundown they might be the source of infections.

In South Africa, it is estimated that about 10.5 million people do not have access to proper sanitation facilities, of which 2.15 million people live in the Limpopo Province and 0.6 million in the Vhembe District [8]. One of the DWAF (now Department of Water Affairs) targets under review was to ensure that all schools in the country be provided with water and sanitation services [9]. In 2006, the Limpopo province had four types of sanitary facilities in public schools and these were flush toilets (20%); VIP/Enviroloo (39%); pit toilets (39%) and the bucket system (2%) [9]. This may indicate that there are schools that still do not have proper water supply and sanitation facilities. Therefore, there is a need to undertake the study since it may serve as a source of motivation to learners and thus play an important role in changing their attitude and behavior.

This study was carried out in at selected secondary schools in the Vhembe District in the Limpopo province. The major objective was to assess the Knowledge, Attitude and Practices (KAP) of learners on Water, Sanitation and Hygiene issues in selected rural and urban schools in Vhembe District of the Limpopo Province. The specific objectives were: to understand the knowledge, attitudes and practices of learners towards water, sanitation and hygiene; to assess the availability and reliability of water supply that is used by learners at the selected secondary schools; and to assess the current status of sanitation and hand washing facilities at the selected secondary schools.

## 2. Methods and Materials

### 2.1. Selection of Secondary Schools

The simple random sampling method was used, relying on random numbers to select sample schools from the list of schools which was provided by the Vhembe District Municipality’s Department of Education. Rural schools were also chosen on the basis of whether they qualified for the feeding scheme which caters for schools based in extremely poor areas. A total of eight schools were selected from 145 secondary schools that were located in Vhembe District of which four were from Thohoyandou, an urban area and the other four schools from rural areas (Table 1).

**Table 1:** Sample size and sampling.

Name of school	Location of study site	Total No of learners	40% Sample
Mukhwantheli Sec	Rural	640	256
Movhe Sec	Rural	300	120
Gole Sec	Rural	582	233
Thase Sec	Rural	466	186
Raluswielo Sec	Urban	738	295
Phaswana Sec	Urban	674	270
Thohoyandou Sec	Urban	1,238	495
Marude Sec	Urban	950	380
Total		5,588	2,236

### 2.2. Data Collection

The primary data included personal observations, questionnaires and informal interviews. A total of 2,236 (40% of 5,588) learners were interviewed. The schools were selected from different circuits within the Vhembe district. Information about the physical aspect, which includes the facilities and the status of the water supply system of the school, was also obtained from the principal’s questionnaire. Personal observations were carried at the schools and a checklist was used to record the information on the current status of water supply and sanitation facilities.

### 2.3. Ethical Consideration

Written permission was sought for and granted by the Department of Education, Vhembe District Municipality, to carry out the study. The individual school authorities were also approached to obtain their consent to carry out the study. Lastly, the learners’ consent (no personal identifiers were recorded) was obtained first after explaining the purpose of the study and that they were not obliged to answer any questions which they did not like or were free to terminate the interview at any given time.

### 2.4. Survey Tools

The tools for data collection of the survey were: questionnaires, personal observation and a checklist. A pilot survey was conducted at the selected schools. Questionnaires were designed to elicit responses on the main water supply sources, sanitation facilities, the knowledge and behavior of learners on personal hygiene, waterborne diseases, water supply and sanitation. Personal observations, using a checklist, were used to collect information regarding water supply sources at schools, personal hygiene practices of learners, the status of hand washing areas, the status of construction, operation and maintenance of sanitary latrines at schools. A guideline for conducting in-depth interviews was designed taking into consideration the customs and difficulties that may hinder construction and maintenance of hygienic latrines, hand washing with soap and safe water usage.

### 2.5. Data Processing and Analysis

In order to ensure the quality of the data, each completed questionnaire was manually checked before it could be coded on MS Excel 2007. The data was analyzed using the Statistical Package for Social Science (SPSS 21) with Chi-square test for independence (with Yates’ Correction for Continuity) at a significance level at 95% confidence interval and relative risks (RR). The procedure based on MS Excel was used to determine the confidence interval for proportions at 95% margin of error [10].

## 3. Results and Discussion

### 3.1. General Characteristics of the Study Population: Knowledge, Attitude and Practice

The survey on knowledge, attitude and practices was conducted at eight secondary schools in both rural and an urban area of Thohoyandou. A total of 2,236 learners (grades 8 to 12) were interviewed, of which 34.90 ± 1.98% of the respondents were from rural schools and 65.10 ± 1.98% from urban schools. In terms of gender, 46.30 ± 2.07% % of the respondents were male while 53.70 ± 2.07%) of the respondents were female.

In relation to hygiene, about 91.40 ± 1.16% of the respondents in the study reported that they were concerned about hygiene, of which 53.20 ± 2.07% were always concerned, 40.40 ± 2.03% sometimes concerned and 6.40 ± 1.01% had no concern at all. In terms of practices, most of the respondents reported that they practiced hand washing, especially before eating and after visiting the toilet. 

**Figure 1:**
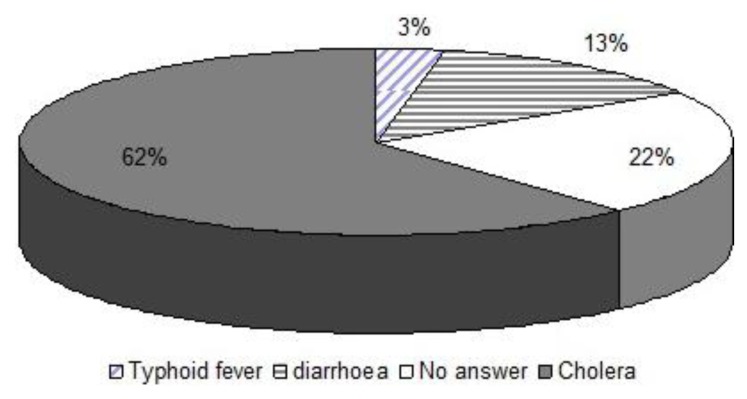
The level of knowledge on waterborne diseases in Vhembe District.

With regard to the behavior of washing fruits before eating them, 81.80 ± 1.60% of the respondents reported that they wash fruits before eating them. The reasons that were advanced were for the removal of bacteria and that the fruits would have passed through many dirty hands before buying them. Removal of dust was also mentioned as another reason for washing fruits before consumption. In terms of knowledge, about 76.80 ± 1.75% of the respondents knew that there are waterborne diseases, even though they could not differentiate between cholera and diarrhoea. They treated cholera as a different disease from diarrheoa while it is not. In terms of knowledge about diarrhoeal diseases, cholera was found to be the most popular waterborne disease among learners, followed by typhoid fever (Figure 1). Apparently 62.5 ± 2.55 % of the respondents from urban schools knew about cholera in comparison to 35.5 ± 2.55% of respondents from rural areas.

Whilst a majority of learners knew about waterborne diseases, a sizeable number (22.0 ± 1.75%) of all the respondents had no knowledge of any waterborne diseases. The survey revealed that most of the respondents who had knowledge about waterborne diseases got it from school, television and radio. However, about 65.0 ± 1.97% did not know about the route of transmission of waterborne diseases, while the others knew that waterborne diseases are mostly transmitted through drinking dirty water. The results are similar to the study of Vivas *et al.* in Angolela district of Ethiopia who found that 60% of the school children did not know the disease transmission routes [11]. In comparison to the study of Noi concerning KAP of children in Vietnam, the waterborne diseases that were known by the respondents were: diarrhoea (62%), parasitic diseases (18.6%), skin diseases (17.6%), eye diseases (11%) and gynecological and obstetrical diseases (3.8%) [12].

### 3.2. The Level of Hygiene and Sanitation Knowledge in Schools

The study indicated that knowledge about water related diseases and its prevention was actually poor in the studied area, with 78.40 ± 1.71% of the respondents admitting that they had no idea on what was meant by water related diseases. Most of those who knew about the prevention of water diseases mentioned that to avoid water based infections such as bilharzia one has to avoid swimming in dirty water. Also the respondents mentioned that water from a stream must be boiled and cooled before drinking to avoid getting ill due to waterborne diseases.

The prevalence of pit latrines was lower in urban schools than in rural schools as indicated by the chi-square test for independence (with Yates Continuity Correction) which indicated a significant association between the type of toilet systems and location of schools (relative risks = 0.39, 95% confidence interval from 0.39 to 0.42). For pit latrines, these were exclusively located in the rural schools (100%) but the urban schools had at least one pit latrine toilet. The presence of pit latrines in the urban areas may be a back up in the event there is no water to use in the flushing toilets. Only schools using flushing toilets had water taps inside their toilets and two other water taps on the school premise. This enabled learners to wash their hands immediately after coming out of the toilet. The other two schools used pit latrines with no taps inside and outside the toilets, which led to learners having to use one tap located at the central point of the school. This might discourage the learners from washing their hands since the tap was far and at times the tap was over crowded. All the schools in the rural areas were found to be using pit toilets for sanitary purposes, without a water tap inside and outside the toilet for washing hands. The non-availability of flushing toilet systems in the rural areas might be linked to the lack of water.

The findings showed that a 100% of the schools had a toilet facility but differed in the technology used. The majority (75%) of the schools had access to pit latrines, whilst 25% of the schools (mostly from urban areas) had access to flush toilet facility. The study by Noi showed that only 73% of the schools had access to pit latrines [12]. The schools in Vhembe district that were using pit latrines had one water tap which was mostly located at the central point of the school. This might probably have a negative impact on the status of the learners’ health because they normally visit the toilet in large numbers during break time, with only one water tap located at the center of the school making it unlikely for all learners coming from to wash their hands at the single water tap. 

In all the schools, it was found that there was no soap provided for hand washing nor were learners encouraged to bring their own soap. About 78.90 ± 1.69% of the respondents felt that teachers were not doing enough in terms of informing learners about practicing safe hygiene and sanitation. The main reason given was that the state of their toilet was generally clean, but in other schools the toilets were dirty and this might discouraged the learners from using the toilets. It is further recommended that the walls of the toilets in schools be decorated or painted with messages that promote sanitation and hygienic practices. This will act as a constant reminder to learners to always practice safe disposal of excreta and the washing of hands after visiting the toilet. On the other hand it will also remind teachers to emphasize the importance of such practices to learners. This will also help to enhance the behavior and practices of learners towards hygiene and proper use of their toilets. Some of the reasons given were that teachers never talked about it which meant that they never cared about such a practice, whereas 21.10 ± 1.69% of the learners felt that teachers were doing enough in informing them, especially during life orientation lessons. The findings are in agreement with the study of Al-Medhawi *et al.* about the use of soap for hand washing in that, householders used less soap for most of the times [13]. The use of soap has the added benefit of reducing childhood diarrhoea [3]. The study of Curtis and Cairncross had shown that washing hands with soap has positive results such as reduction in diarrhoeal diseases (43%) and diarrhoea with life threatening conditions (47%) [14]. The results of this study recommend that the education authorities should increase the budget for sanitation in schools to enable the purchase of soap and disinfectants.

With regard to acquiring information, learners believed that it was the responsibility of the government, school and parents to ensure that learners are aware of methods of practicing safe hygiene and sanitation. The learners seemed to be contradicting between a general source of water and a clean source of water, only 46.6 ± 2.07% knew at least one source compared to the 54.4 ± 2.07% who did not know any source of clean water. This was found to be surprising because one would expect the learners at secondary school to be knowledgeable about the fact that a river is a source of water but not necessarily a clean source, since it is not treated. The study revealed that municipal water was found to be the most popular source of clean water followed by borehole and bottled water respectively.

The study revealed that the level of hygiene knowledge for urban schools was high (71.70 ± 2.04%) in comparison with rural schools (28.30 ± 2.04%), with of the respondents saying that they acknowledged the importance of washing hands after visiting the toilet. They did so mostly to remove germs and bacteria while on the other hand preventing diseases.

**Figure 2:**
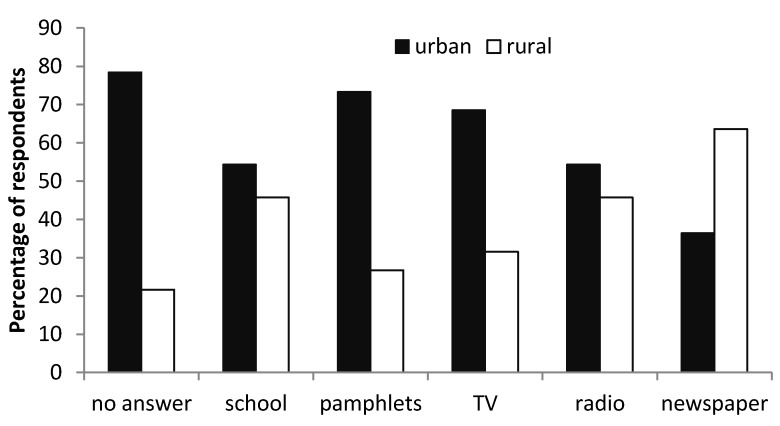
The different sources of information on waterborne disease.

Regarding sanitation, about 76.7 ± 1.75% of the respondents knew at least one waterborne disease while the other 23.2 ± 1.75% had no clue as to what was meant by waterborne disease. There was disparity in the level of knowledge about waterborne diseases with 60.7 ± 2.31% of the respondents from urban schools were more knowledgeable about waterborne disease in comparison to 39.3 ± 2.31% of respondents from rural schools. In the study by Noi, at least 30.4% of the respondents could not name any waterborne diseases [12]. Schools and television proved to be the most popular sources of information when it comes to waterborne diseases (Figure 2). This was probably because learners spend most of their time at school and when they got home, they spend most of their time watching television. Also there were disparities between the rural and urban schools, with rural schools obtaining most of their information from newspapers.

A majority of the respondents in this case did not know anyone who had been affected by a waterborne disease. For rural schools, only 39.3 ± 2.31% of them knew or had come across someone who had been affected by a waterborne disease, while in urban schools, 60.7 ± 2.31% of respondents knew somebody. They also knew the transmission route for waterborne diseases was through drinking water that is contaminated with fecal matter from animals, but in their case they were not certain whether it was water related or not. The majority of the respondents had no knowledge when it comes to water based diseases and its prevention (78.4 ± 1.71%).

### 3.3. The Level of Hygiene and Sanitation Attitude in Schools

**Figure 3:**
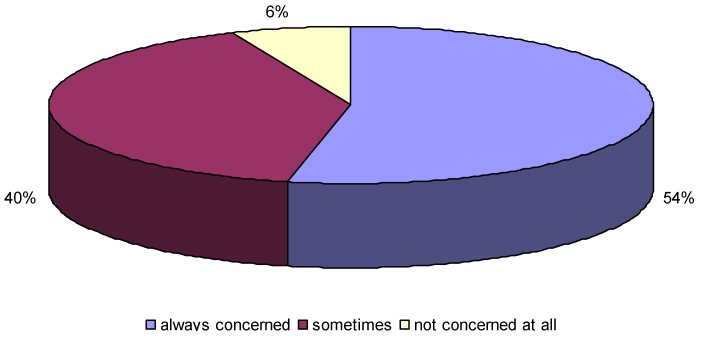
The level of attitude on hygiene and sanitation in schools.

The concern about hygiene was found to be high (Figure 3). A majority of this group felt that the government should play a leading role in their development as they believed that it was government’s responsibility to ensure that they were aware of issues surrounding the practices of safe hygiene and sanitation. Others felt that parents and the school should also take the responsibility of ensuring that learners were aware of the benefits of practicing safe hygiene and sanitation.

A chi-square test for independence (with Yates Continuity Correction) indicated a significant association between the concern about the hygiene and location of schools showed that urban schools were more concerned about hygiene (relative risks = 0.89, 95% confidence interval from 0.81 to 0.98). For concern about hygiene between the urban and rural schools was variable, 63.8% (urban) and 36.2% (rural) but was above 90% within the schools (rural or urban). This means that they regarded hygiene as a very important aspect of human life because most learners were always concerned, some were sometimes concerned and a few of them not concerned at all.

**Figure 4:**
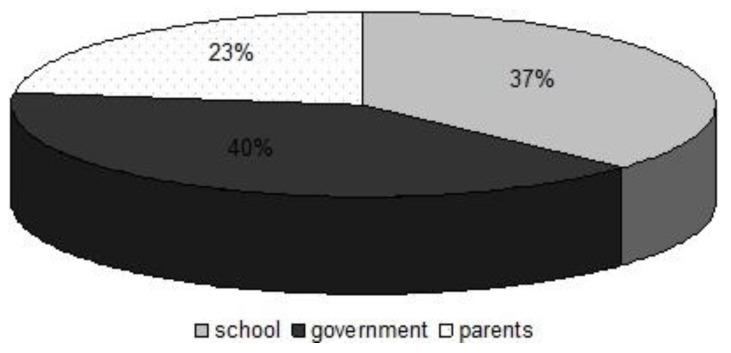
The responsibility of teaching safe hygiene and sanitation practices.

Most learners in this category believed that it was the responsibility the government to ensure that they know of issues concerning the practice of safe hygiene and sanitation. This may also be attributed to poorly maintained toilet systems. A dirty toilet discourages learners from using it and they might opt for the bush as an alternative. They believed that the government and the schools should play a leading role as far as hygiene promotion is concerned, while on the other hand parents were less considered as important role players in hygiene promotion to learners (Figure 4).

### 3.4. The Level of Hygiene and Sanitation Practice in Schools

The study indicated that the level of hygiene and sanitation practices was high in schools. A chi-square test for independence (with Yates Continuity Correction) indicated a significant association between the washing of hands before eating and location of schools showed that urban schools were more concerned about washing of hands (relative risks = 0.62, 95% confidence interval from 0.55 to 0.69). For washing of hands between the urban and rural schools was variable, 70.3% (urban) and 29.7% (rural) but was above 65% within the schools (rural or urban). Those who practiced hand washing pointed out that they did mostly before eating and after visiting the toilet. This practice was mostly affected by the fact that water was not always available in some rural schools. This could be supported by the fact that in one of the schools it was found that, the rural school had no access to clean drinking water so it was forced to buy water from a nearby village where there was a private borehole. The purchased water would then be stored in storage tanks and once finished they would purchase more which led to the school being without water for several days while awaiting another delivery. A chi-square test for independence (with Yates Continuity Correction) indicated no significant association between the washing of fruits before eating and location of schools showed that urban schools were more concerned about washing of fruits (relative risks = 1.05, 95% confidence interval from 0.97 to 1.15). For washing of fruits before eating between the urban and rural schools was variable, 65% (urban) and 35% (rural) but was above 80% within the schools (rural or urban). More than three quarters of the respondents agreed that they did wash fruits before consumption for reasons including the removal of bacteria and that the fruits were handled by many different people. The other reason was for the removal of dust particles from the fruits. The reasons for not washing the fruits were lack of water at the fruit market place; the water tap was far away, the fruit looked clean and laziness.

The studies of O’Reilly *et al.* and Blanton *et al.* involving students in western Nyanza District of Kenya showed similar patterns as ours that the majority of the students/learners washed their hands before eating and after using the toilet [15,16]. A series of quick impact initiatives, such as drama and music, aimed at encouraging hand washing with soap at school level, should be introduced. This will help create awareness on the subject while enhancing the learners’ knowledge as far as hygiene practice is concerned. This may also help in converting their knowledge and attitude into proper actions as they compete against each other for the prizes at stake. This should be targeted for the celebration of the Global Hand washing Day in October of every year.

A chi-square test for independence (with Yates Continuity Correction) indicated no significant association between the washing of hands before eating and gender (female or male), (relative risks = 1.01, 95% confidence interval from 0.97 to 1.05). For washing of hands before eating between the gender was variable, 46.5% (male) and 53.5% (female) but was above 78% within the gender (male or female). Further analysis indicated that the differences between male and female learners in practicing good hygiene were minimal. They all washed their hands mostly before eating and after visiting the toilet. A chi-square test for independence (with Yates Continuity Correction) indicated a significant association between the washing of fruits before eating and gender (female or male), (relative risks = 0.90, 95% confidence interval from 0.86 to 0.93). For washing of hands before eating between the gender was variable, 43.7% (male) and 56.3% (female), but was above 77% within the gender (male or female). The behavior of learners in terms of practices differs by less than 10%, with the girls always having a high percentage of practice than the boys.

### 3.5. Availability of Water Supply for Drinking and Sanitation in Schools

**Figure 5:**
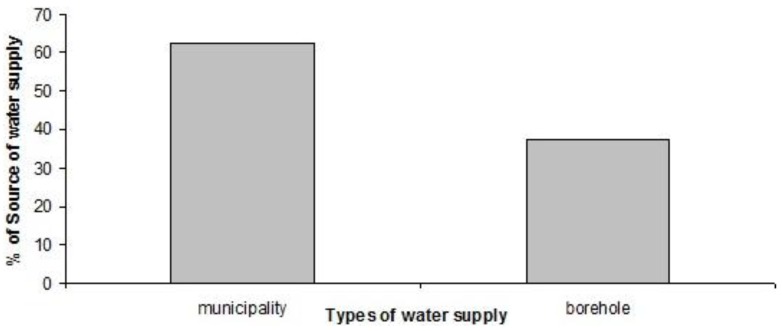
Sources of Water supply for schools.

All the schools from urban areas obtained water of good quality on a regular basis from municipal supply while in the rural areas the three of the four schools used borehole water. Mukhwantheli Secondary was the only rural school that was using a municipal water supply (Figure 5).

The rural schools used Jojo storage tanks to store water that was abstracted from the borehole and also to allow for accessibility and use of the water. In all the rural schools, the only tap available was located at the central point of the school. In terms of water shortages, urban schools did not experience water shortages while some rural schools experienced shortages, but normally those shortages lasted less than a week. Other rural schools experienced water shortages on a regular basis. It was found out at one of the schools that the school had no access to clean drinking water and as a result was forced to buy water from a nearby village where there was a private borehole. Even though the water looked clear, the quality, in terms of microbial contamination, was unknown and this was a big concern among school authorities. The studies of Noi, O’Reilly *et al.* and Vivas *et al.* have reported similar sources of drinking water and water for sanitation purposes ranging from municipal water, dug wells, rain water, surface water sources (rivers, lakes and ponds) and boreholes [11,12,15,17].

### 3.6. Field Observations for Both Rural and Urban Schools

**Figure 6:**
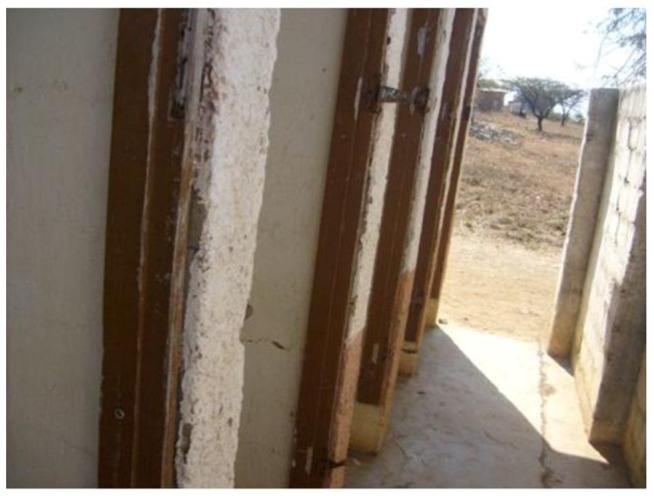
Sanitation facilities showing absence of doors.

Schools generally had sufficient toilet facilities except that the maintenance was poor and had similar findings to the study of Bility and Onya [17]. In all the schools it was found that there were no sanitary bins (pads) for girls. In some instances toilets, especially in rural schools, had broken doors and did not provide the required privacy (Figure 6). The issue of privacy and lack of sanitary bins is important to the girl child as demonstrated by the review paper by Jasper *et al.* that reported high absenteeism of the girl child [18].

Although there was 100% coverage of hand washing facilities in the schools, there was a considerable disparity in the number and location of these. Only 25% of the schools had hand washing areas that were located inside the flush toilets, although there was no soap provided. The remainder of schools (75%) had hand washing facilities (one tap, no soap) that were located at the centre of the schools and were about 100 m from the toilet. These findings are an improvement in comparison to the Viet Nam where 29% of schools had access to hand washing facilities with sufficient water [12]. Furthermore, the study from Viet Nam showed that 4.6% of schools had soap at the hand washing facility [12], while there was none in this study.

The only water-related disease ever reported was bilharzia in two of the rural schools (Gole and Thase) and it was detected in about eight learners by the local clinic. Only three, Gole, Thase and Thohoyandou secondary, of the eight schools, promoted sanitation issues as part of the school curriculum. Here, learners were always made aware of using clean water and toilets. An observation from those schools was that they used charts from the Department of Health, which were displayed in all classrooms, to promote hygienic practices. In most cases, authorities revealed that learners were tasked to clean the toilets at the schools, as was the case, especially in rural schools, and only one school used volunteers from their community to clean their toilets. The only disinfectant used was found to be Jay’s fluid, which contains liquid chlorine.

All the schools did not observe the Global Hands Washing Day, in fact they did not have a clue that such a day existed in the month of October. About five schools felt the government departments were not doing enough to assist them on hygiene and sanitation promotion. The Department of Health and Social Development for instance did not give schools promotional material such as posters without them asking for such materials, they only gave posters if requested, which gave the authorities the mentality that the department did not recognize the importance of promoting safe hygiene and sanitation in schools.

## 4. Conclusions

A determination of the level of knowledge, attitude and practices on hygiene led to the conclusion that learners in the study area have sufficient knowledge about safe hygienic practices. It however, seems that the knowledge is not properly utilized as numerous field visits confirmed that maintenance of sanitary systems in schools was actually poor. In terms of the questionnaire’s results, the attitude and practices were also found to be high among learners while numerous field visits indicated that there is lack of knowledge and practices. The lack of maintenance in sanitary facilities indicates that school authorities were ignoring the importance of using clean toilets. This contradiction might have been caused by the fact that learners knew, for instance the importance of washing hands after visiting the toilet, therefore they could not tell the interviewer something that would make them feel ashamed of themselves. The observations during numerous field visits indicated that only one school from rural areas had adequate sanitation facilities which were well maintained, while in the urban schools sanitation facilities were adequate except that they were not properly maintained. Only two schools from the urban part had proper hand washing facilities. Most school authorities felt that the water supply was inadequate and they had to force learners to clean their own toilet because there was no one employed to take care of such activities. This might be the main reason why the toilets in most schools were always dirty, as learners could not voluntarily perform such activities and no one was set to enforce it from the school authority.
